# Book Review: Amebiasis. Biology and Pathogenesis of *Entamoeba*

**DOI:** 10.3389/fmicb.2016.00819

**Published:** 2016-05-27

**Authors:** John P. Ackers

**Affiliations:** Department of Pathogen Molecular Biology, Faculty of Infectious and Tropical Diseases, London School of Hygiene and Tropical MedicineLondon, UK

**Keywords:** *Entamoeba histolytica*, amebiasis, review, *Entamoeba histolytica* biology, amebiasis pathogenesis

In every research laboratory there is “the book”—the standard text, given to people newly entering the field and frequently referred to by the rest of us. In the amoebiasis world, this was the massive and magisterial 1988 volume edited by Jonathan Ravdin. But since then there has not been a comparable work produced which would reflect the enormous increase in knowledge which has accrued in the subsequent 25 years. Is this newly-published book it?

Part I deals with four topics. The first chapter describes the continuously expanding *Entamoeba* universe; a multi-branched (phylogenetic) tree which has grown explosively from the seed planted 20 years ago by the realization that most people excreting cysts of “*Entamoeba histolytica*” were in fact infected with *Entamoeba dispar*. Cheap and rapid DNA sequencing has driven this revolution; but while raw data may now appear in terabytes, assembling it into an accurate genome has proved challenging and frustrating, as chapter 2 makes clear. The next two chapters cover genetic typing of isolates; this may be less computationally taxing than genome assembly but the results are equally hard to interpret. Genetic manipulation has been an immensely powerful research technique in many fields; disappointingly it has turned out to be difficult to apply to *E. histolytica*. Much useful data has been accumulated but many frustrations remain, as the final chapter makes clear.

Part II considers the regulation of gene expression. The first chapter deals with mapping the transcriptome; written in a clear and approachable style, this chapter is a model of what an overview should be. The organization of *E. histolytica* ribosomal RNA genes is somewhat unusual and their structure and replication is clearly explained in Chapter 8. The following chapter covers the regulation of gene expression by small RNA molecules and expands on some of the topics dealt with in Part I. Chapter 10 deals with retrotransposons—mobile genetic elements which make up a significant fraction of most eukaryote's DNA, including *E. histolytica*. The authors are from one of the laboratories which have contributed most to this field and their expertise is evident. The final chapter focusses on the links between environmental stress and epigenetic regulation—a topic attracting great interest amongst both basic scientists and clinicians.

Part III covers Cell Biology and Signaling. The first chapter details the many steps between initial recognition of an ingestible particle and its final destination. Many different molecules are involved, listed in a useful summary Table. Chapter 13 covers signaling. Evolution doesn't favor simplicity and nowhere is this more apparent than the interlocking tangle of eukaryotic signaling pathways. This chapter briefly describes the major paths through a forest of acronyms. The next chapter is more specialized, dealing with a newly discovered family of transmembrane kinases, and is followed by a review of one of the most active areas of current research—cell surface molecules as virulence determinants. A well written chapter which clearly summarize how much we now know—but how much more there is still to understand. The remainder of Part III covers cell division (a seriously odd process, even by the standards of *E. histolytica*), trafficking of cysteine proteases and mitosomes. The essential functions of these mitochondrial relics differ from organism to organism; those of *E. histolytica* are well described here.

Part IV is concerned with with Metabolism. The opening chapter describes the metabolomics analysis of *E. histolytica*. The potential of the method is clear and already surprising and exciting results have emerged. The remaining chapters are more specialized. Chapter 20 deals with glucose metabolism and its investigation by Metabolic Control Analysis and the following two chapters cover the proteins involved in the biosynthesis of cysteine and DNA replication respectively.

Part V concentrates on Pathogenesis and Immunity. Chapter 23 describes the pathology seen in patients with the commonest forms of clinical amoebiasis. A brief summary of the many steps involved in pathogenesis is followed by a description of the available animal models. Continuing with the attack, Chapter 25 describes the contribution of cysteine proteases to pathogenesis. Tuning to the host's attempts to defend itself, three chapters (rather oddly arranged) discuss this. Chapter 24 and 26 cover innate defenses and in particular the vital role of intestinal mucus—if this is not penetrated there is no pathology. Chapter 28 covers the links between host genetic makeup and innate susceptibility, while Chapter 29 discusses an equally important topic—the adaptive immune response produced in infected humans and animal. Finally, Chapter 26 discusses in detail a point made by several others—that the hosts immune response is itself responsible for a significant amount of the tissue destruction observed.

The last part of the book covers drug discovery and drug resistance. This section begins with an excellent and informative chapter on metronidazole. One really surprising fact is that although clinical resistance has appeared amongst most of the organisms against which metronidazole is employed, such resistance has never been observed with *E. histolytica*. Nevertheless, there is still much interest in developing alternative chemotherapeutic agents, and this is the subject of the final chapters in the book.

The book is in general well produced although some of the Figures and Tables (reproduced from other sources) are really too small; for example Figures 8.1, 9.1, and 14.1. I noticed a small number of proof-reading errors—cross references are missing from Chapter 12 and on page 531 “ESR” is, here, not an abbreviation for “Erythrocyte Sedimentation Rate.”

These days any book that attempts to summarize a fast-moving area such as research on *E. histolytica* is out-of-date before it goes to the printer. This volume is no exception, but it is as current as could be expected. It focusses on specific areas; others such as clinical amoebiasis or epidemiology are not dealt with in any detail, but the title of the volume makes it clear that this is not its remit. This is principally a handbook for laboratory researchers and for them it will prove invaluable for many years to come.

## Author contributions

The author confirms being the sole contributor of this work and approved it for publication.

### Conflict of interest statement

The author declares that the research was conducted in the absence of any commercial or financial relationships that could be construed as a potential conflict of interest.

